# Red and Yellow Injectable Platelet-Rich Fibrin Demonstrated Differential Effects on Periodontal Ligament Stem Cell Proliferation, Migration, and Osteogenic Differentiation

**DOI:** 10.3390/ijms21145153

**Published:** 2020-07-21

**Authors:** Prakan Thanasrisuebwong, Sirichai Kiattavorncharoen, Rudee Surarit, Chareerut Phruksaniyom, Nisarat Ruangsawasdi

**Affiliations:** 1 Dental Implant Center, Dental Hospital, Faculty of Dentistry, Mahidol University, Bangkok 10400, Thailand; Prakan.tha@mahidol.ac.th; 2 Department of Oral and Maxillofacial Surgery, Faculty of Dentistry, Mahidol University, Bangkok 10400, Thailand; Sirichai.kia@mahidol.ac.th; 3 Department of Oral Biology, Faculty of Dentistry, Mahidol University, Bangkok 10400, Thailand; rudee.sur@mahidol.ac.th; 4 Department of Pharmacology, Faculty of Dentistry, Mahidol University, Bangkok 10400, Thailand; chareerut.phr@mahidol.ac.th

**Keywords:** injectable platelet-rich fibrin, periodontal ligament stem cells, bone regeneration, osteogenic differentiation, cell proliferation, cell migration

## Abstract

The biological benefits of using two fractions derived from injectable platelet-rich fibrin (i-PRF) in bone regeneration remain unclear. Thus, the current study examined two fractionation protocols producing yellow i-PRF and red i-PRF on periodontal ligament stem cells (PDLSCs). The i-PRF samples from five donors were harvested from two different levels, with and without a buffy coat layer, to obtain red and yellow i-PRF, respectively. The PDLSCs were isolated and characterized before their experimental use. The culture medium in each assay was loaded with 20% of the conditioned medium containing the factors released from the red and yellow i-PRF. Cell proliferation and cell migration were determined with an MTT and trans-well assay, respectively. Osteogenic differentiation was investigated using alkaline phosphatase and Alizarin red staining. The efficiency of both i-PRFs was statistically compared. We found that the factors released from the red i-PRF had a greater effect on cell proliferation and cell migration. Moreover, the factors released from the yellow i-PRF stimulated PDLSC osteogenic differentiation earlier compared with the red i-PRF. These data suggest that the red i-PRF might be suitable for using in bone regeneration because it induced the mobilization and growth of bone regenerative cells without inducing premature mineralization.

## 1. Introduction

Bone regeneration in dentistry is a dynamic approach for treating critical size bone defects that are unlikely to self-heal. These complicated defects require a triad of tissue engineering, cells, signaling molecules, and a scaffold. Fibrin is a natural scaffold that has been used clinically to promote bone regeneration due to its outstanding biocompatibility, manageable biodegradability, and ability to deliver signaling molecules [1,2]. Fibrin proteins can be obtained from pooled plasma, which can be promptly used, but can transmit viruses, or a patient’s own plasma, which is safe and biocompatible; however, obtaining it from the patient may be painful and requires multistep preparation [3].

Platelet-rich fibrin (PRF), introduced by Choukroun et al. [4], is an autogenous fibrin product that, unlike platelet-rich plasma, can be obtained from blood using low-speed centrifugation without adding anticoagulants. The benefits of using PRF for bone regeneration include that PRF functions as a scaffold for endogenous cell homing and as a signaling protein reservoir for osteoinduction [5]. PRF was initially used as a membrane, which enhanced soft tissue healing rather than bone regeneration [6]. Subsequently, the development of injectable PRF (i-PRF) allowed its use in bone tissue regeneration by not only enriching the growth factors in bone substitute materials, but also allows the bone graft particles to stick together for better handling [7]. 

Clinicians use i-PRF in combination with particulate bone grafts to promote the biological and physical properties of the materials [8]. The combination has shown benefits, such as improved angiogenesis and handling ability by combining the small bone granules into a bulk material to use in bone grafting surgery [9,10,11]. Further use of i-PRF demonstrated that it was effective in regaining gingival thickness, reducing periodontal pocket depth, and attachment loss in periodontal tissue regeneration [12,13]. Although a few cases have reported an encouraging outcome, there has been a paradigm shift concerning the use of i-PRF in clinical bone regeneration.

The i-PRF preparation methods vary based on centrifuge characteristics and centrifugation speed, and the different fractions from different areas based on the junction between the red blood cell and the enriched fibrin plasma layers. Although Wang et al. [14] reported harvesting the upper layer of whole blood following centrifugation, their protocol produced a yellowish i-PRF; our previous study used red i-PRF, which was harvested at the interface with the buffy coat layer. The red i-PRF contains a greater number of cells and platelet-derived growth factor (PDGF). In contrast, the yellow i-PRF, which was harvested from the upper yellow zone above the junction, resulted in better fibrin clot formation and strength [15]. The handling properties between the types are not clinically meaningful, and similar amounts of vascular endothelial growth factors (VEGF) and transforming growth factor-beta 1 (TGF-β1) are released [15]. The cell responses in bone engineering, however, might be different because the red i-PRF released a greater amount of cell signaling molecules related to mesenchymal stem cell motility, growth, and differentiation. However, the effects of the growth factors released from each iPRF type on human periodontal ligament stem cells are currently unknown.

To determine which i-PRF fraction has the greatest effect on human periodontal ligament stem cells osteogenic differentiation, the aim of this study is to evaluate the effect of i-PRF on mesenchymal stem cell behavior relating to the process of mineralized tissue formation. The two i-PRF fractions were tested using periodontal ligament stem cells (PDLSCs), which are mesenchymal stem cells that can differentiate to the osteoblastic lineage, and are involved in bone tissue regeneration [16]. We hypothesized that the red i-PRF would have a better effect on PDLSC behavior. Thus, the effects of red vs. yellow i-PRF on PDLSC proliferation, migration, and osteogenic differentiation were evaluated in this study.

## 2. Results

### 2.1. PDLSC Isolation and Characterization

**Figure 1:**
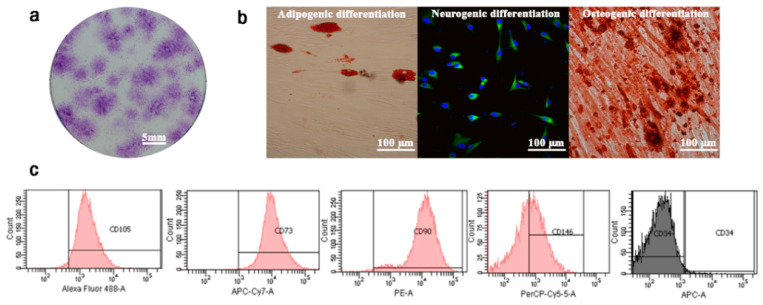
Periodontal ligament stem cell characterization. (**a**) The isolated cells were evaluated for single colony formation (scale bar = 5 mm), (**b**) and multi-lineage differentiation potency; adipogenic differentiation, neurogenic differentiation, and osteogenic differentiation, (scale bars = 100 µm). (**c**) Analysis of the stem cell markers showed 70‒99% CD105^+^, CD90^+^, CD73^+^, CD146^+^, and CD34^−^ cells.

The cells isolated from the periodontal ligament demonstrated the main mesenchymal stem cell characteristics. Their morphology was spindle-shaped, similar to fibroblasts, in culture. At a low cell-seeding number, the cells formed single colonies that stained with Giemsa (Figure 1a). Further assays demonstrated the multi-lineage differentiation potential of the isolated cells (Figure 1b). Flow cytometry revealed high mesenchymal stem cell marker expression of CD73 (99.8%), CD90 (99.8%), CD105 (83.8%), and CD146 (31.3%), and scant CD34 expression (0.2%) (Figure 1c). 

### 2.2. Proliferative Effects on PDLSCs 

**Figure 2:**
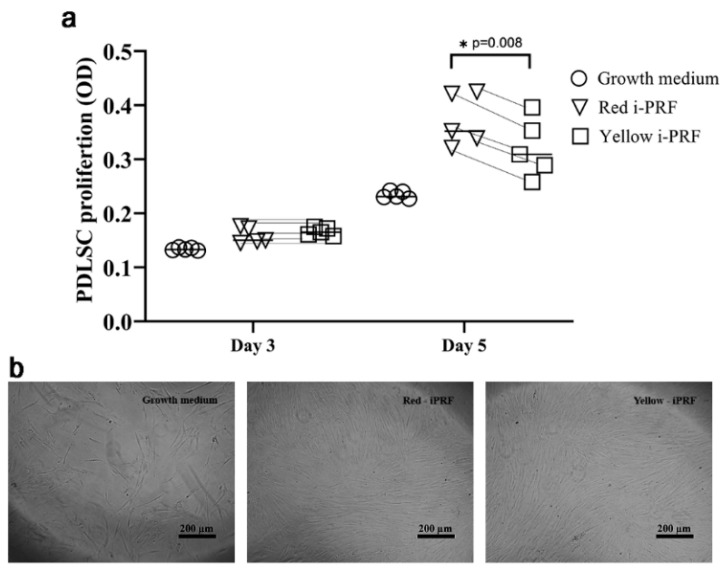
Proliferative effects on periodontal ligament stem cells. (**a**) Cell culture medium containing the release from the red i-PRF enhanced cell proliferation significantly greater than the medium consisting of the release from the yellow i-PRF at day 5. (**b**) Live cell images of cell number in the different culture mediums (scale bars = 200 µm). Significance was assessed using one-way ANOVA followed by post hoc least-significant difference tests to compare the differences between groups. The data are shown as individual donor pairs. * *p* < 0.05.

The effects of the yellow and red i-PRFs on cell proliferation were investigated at day 0, day 3, and day 5. The number of PDLSCs gradually increased in every group over the evaluation period. During the first three days, the cell number between two i-PRF groups and the growth medium group was similar. However, we observed that PDLSCs treated with growth medium containing the released growth factors (the release) from the red and yellow i-PRFs exhibited a greater number of cells after five days compared with cells treated with growth medium (Figure 2). Moreover, cell proliferation in the red-iPRF group was significantly higher than that of the yellow i-PRF group (*p* = 0.046).

### 2.3. Trans-Well Migration 

**Figure 3:**
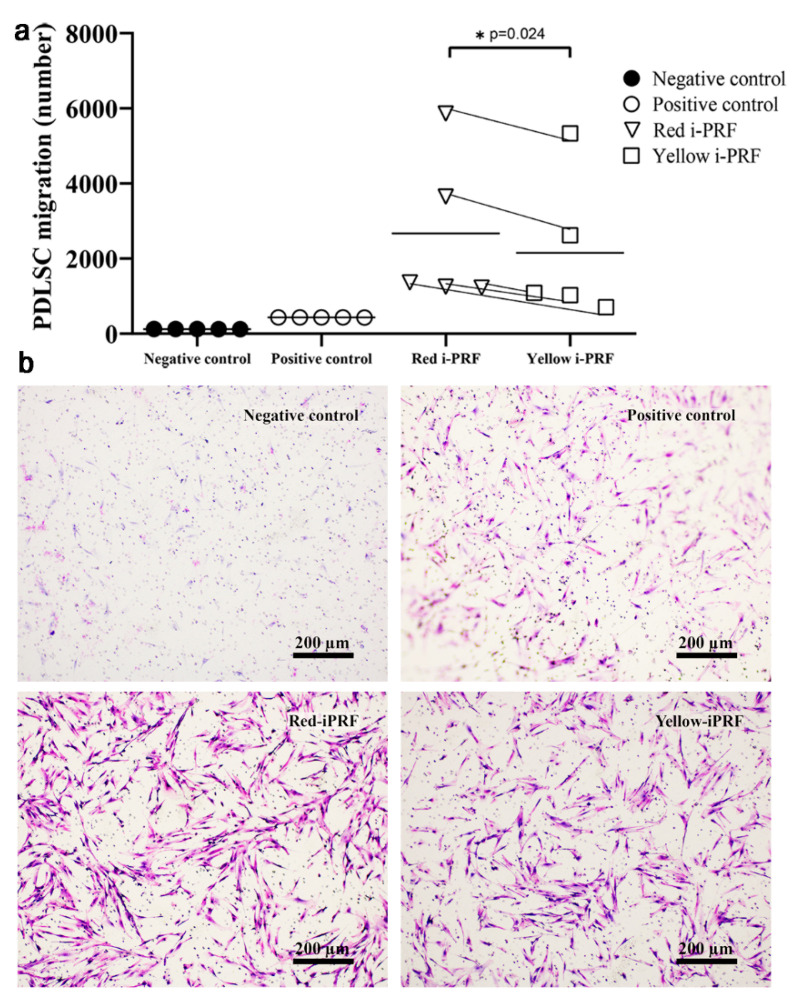
Migration of periodontal ligament stem cells. Conditioned medium obtained from the release from red or yellow i-PRFs was loaded into the growth medium supplemented with fetal bovine serum as the positive control. The medium without any supplement was the negative control. The number of migrated cells was significantly higher in the red i-PRF group than the yellow i-PRF group as shown (**a**) quantitatively and (**b**) microscopically (scale bar = 200 µm). Significance was assessed using one-way ANOVA followed by post hoc least-significant difference tests to compare the differences between groups. The data are shown as individual donor pairs. * *p* < 0.05.

The cell migration assay was performed using a Boyden Chamber loaded with the i-PRF conditioned medium in the lower chamber. Although the PDLSCs migrated into the lower chamber after 24 h in all experimental groups, the i-PRF groups demonstrated greater cell migration compared with the positive control (basal medium with FBS) (Figure 3a). Adding the release from the red i-PRF attracted significantly more cells to the lower chamber compared with the release from yellow i-PRF (*p* = 0.024). In contrast, the negative control group (α-MEM medium without any supplement) presented the lowest number of migrated cells (Figure 3b).

### 2.4. Osteogenic Differentiation 

**Figure 4:**
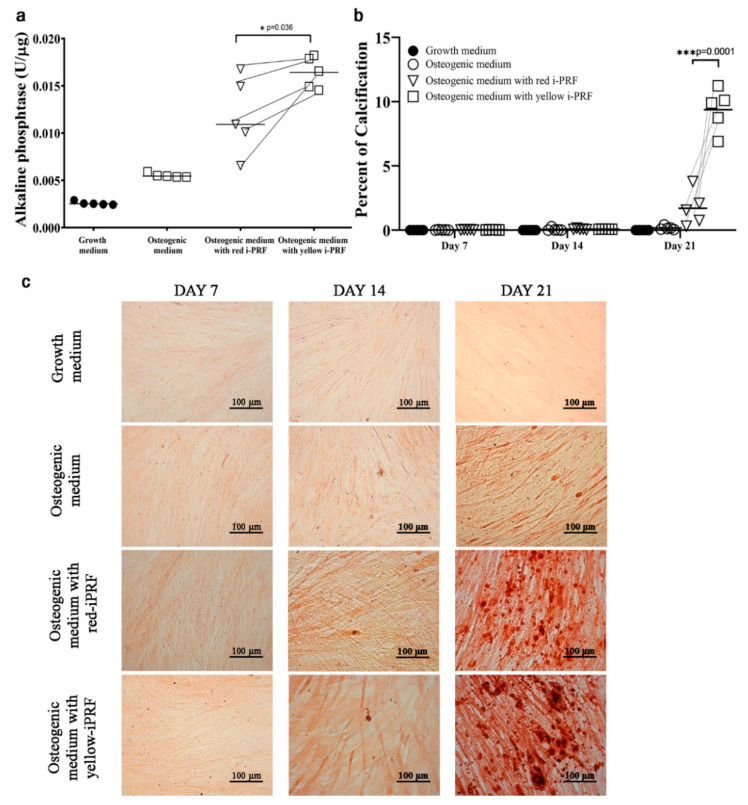
Osteogenic differentiation of periodontal ligament stem cells. Effects of the release from the i-PRF types on osteogenic differentiation were tested in parallel with osteogenic medium as the positive control and growth medium as the negative control. The release from the yellow i-PRF improved osteogenic differentiation more than the release from the red i-PRF demonstrated a significantly higher (**a**) alkaline phosphatase activity, (**b**) percent of calcification and (**c**) alizarin red staining (scale bar = 100 µm). Significance was assessed using one-way ANOVA followed by post hoc least-significant difference tests to compare the differences between groups. The data are shown as individual donor pairs. * *p* < 0.05 and *** *p* < 0.001.

PDLSCs were cultured in osteogenic medium containing the release from red and yellow i-PRF for 21 d to evaluate their osteogenic differentiation. After 3 d, the alkaline phosphatase (ALP) activity of the PDLSCs treated with osteogenic medium increased more than the cells treated with growth medium (Figure 4). However, the 20% i-PRF conditioned medium resulted in greater osteogenic differentiation compared with the control groups. Higher ALP activity was found in the PDLSCs treated with osteogenic medium plus yellow i-PRF compared with adding the red i-PRF (*p* = 0.036). Mineralization by differentiated PDLSCs was detected after 21 d, and alizarin red staining of the calcified foci correspondingly showed that the staining became stronger over time when the cells were treated with osteogenic medium. The image measurements revealed that both i-PRFs generated a higher percent calcification compared with the control groups at day 21. Additionally, using the yellow i-PRF conditioned medium significantly increased the percent calcification compared with the red i-PRF (*p* = 0.0001). 

## 3. Discussion

PRF is an autologous biomaterial derived from blood after being briefly centrifuged at a low speed and without adding an anticoagulant. Three layers are subsequently produced: a bottom layer of red blood cells; an intermediate layer enriched with platelets, fibrin polymer, white blood cells, and plasma protein; and an upper layer of platelet-poor plasma. The i-PRF is collected from the upper liquid layer [17], which is not clearly separated into three layers as with a PRF membrane. A study collected the plasma 2 mm above the junction for i-PRF [5], while others used the plasma from as close as possible to the red blood cell layer [9,18]. Mourao et al. found that there is the intermediate orange layer, which contained more platelets and white blood cells [19]. Although the intermediate layer is not always observed, our previous study demonstrated that the red i-PRF collected at the junction, where the buffy coat may be present, contained more platelets and blood cells that released greater amounts of growth factors, but less fibrin scaffold, compared with the yellow i-PRF collected 1 mm above the junction [15]. It is currently unresolved whether red i-PRF is better than yellow i-PRF for use in bone regeneration. Therefore, the current study used two fractionation protocols producing yellow i-PRF and red i-PRF based on the fraction of the centrifuged plasma above and at the junction between the red blood cells and yellowish plasma, respectively. The results confirmed our hypothesis that the released growth factors from the red i-PRF would have better effects on cell proliferation and cell migration. In contrast, the release from the yellow i-PRF stimulated PDLSC osteogenic differentiation earlier compared with the red i-PRF. 

Growth factors are crucial for promoting osteogenesis and neo-angiogenesis by recruiting mesenchymal stem cells and inducing cell differentiation. The growth factors previously found in i-PRF are VEGF, PDGF, and TGF-β1, which can either directly induce new bone formation or indirectly promote bone healing [15,20]. The bone-morphogenic protein-2 release profile from i-PRF was inferior compared with a natural blood clot, but exhibited a better correlation between protein level and mineralization [21]. In contrast, the accumulated release of insulin-like growth factor-1 and epidermal growth factors presented a significantly higher total release than platelet-rich plasma product after 10 d [17]. Although biochemical signals are released from i-PRF, their effects on cells are limited and, surprisingly, not concentration-dependant [21]. Several studies found a wide range of biocompatibility using 20‒100% conditioned media from i-PRF on human fibroblasts [17] and osteoblasts [8,21,22]. Optimal cell viability also varied over 1‒5 d [17,21]. Moreover, PRF membranes demonstrated high chemotactic activity and mineralization by dental pulp stem cells at a 20% dilution of the concentrated growth factor medium obtained similar to our protocol [23]. This is the first study to demonstrate the effects of i-PRF on mesenchymal stem cells derived from the periodontal ligament, which are responsible for forming the periodontium and the typical cells homing to an alveolar bone defect [16]. A minimal concentration (20%) of the release after 3 d was used in this study to reduce potential detrimental effects, and the results did not demonstrate cytotoxicity over the culture period.

Prior to optimum wound healing and tissue regeneration, a cocktail of several growth factors is required for a well-orchestrated integration of the complex biological and molecular events of cell homing [24]. An important early step is to attract cells to migrate from adjacent tissue, bone marrow, and circulation to the area of the bone graft to participate in bone regeneration [25]. Cell proliferation is the next required step to increase the number of cells in the bone graft site. The processes of cell migration and proliferation during bone regeneration are strongly regulated by signaling molecules [26]. Our previous study has shown that both i-PRF types continuously released growth factors for 14 d. However, the growth factors at the late time points were abundantly released from the red i-PRF, which was also used for further cell assays in this study [15]. The different biological characteristics of each i-PRF exhibited different biological effects on the PDLSCs. We found that the release from the yellow and red i-PRFs enhanced cell proliferation and migration better than the controls, similar to other studies on different cell types [14,17,21]. However, the red i-PRF demonstrated a greater effect compared with the yellow i-PRF. The growth factor concentration in the release used in this study was measured and presented in our previous report [15], and these results correspond to earlier studies that the red i-PRF, containing more platelets and cells, released more VEGF and PDGF, which are potent stimulators of cell proliferation and migration [15,27,28]. Here, the advantages of leukocytes in the red i-PRF are also noteworthy. The intermediate layer between the yellow plasma and red blood corpuscle layers has the highest concentration of leukocytes [19], whose presence greatly contributed to increased mesenchymal stem cell migration [29]. Likewise, scanning electron micrographs of the red i-PRF has demonstrated greater amounts of leukocyte, platelets, and erythrocytes compared with the yellow i-PRF [15]. Furthermore, in vitro proliferation was found after red i-PRF treatment at day 5, but not at earlier time points. This might be because the percentage cell proliferation was an average from different donors, which might contain varied concentrations of growth factors. The MTT assay also provided information of overall cell growth, rather than about individual cells. Therefore, the red i-PRF might enhance each individual cell at a different growth rate as also described in a previous study [30].

PDLSCs have the capacity to differentiate to osteoblastic-like cells and to form calcium nodules in vitro [31,32]. In our culture protocol, the PDLSCs began to form calcium nodules on day 21. However, the PDLSCs cultured with the release from either i-PRF demonstrated earlier mineralization, and both i-PRFs promoted better osteogenic differentiation compared with the control group. These results agree with those of a previous study where the i-PRFs accelerated calcification of osteoblasts [14]. Compared with their effects on proliferation and migration, the two fractions demonstrated the opposite effects in inducing osteogenic differentiation. ALP activity was greater when PDLSCs were treated with the conditioned medium from the yellow i-PRF, as was calcium deposition on day 21. A possible reason might be that osteogenic differentiation is not regulated by one signal molecule, but requires a cascade of growth factors to induce bone formation. A complex biological signal is likely required in this process at the optimal concentration and time. Although a previous study found that a higher concentration of PDGF was released by the red i-PRF [15], PDGF receptor signaling has been shown to have little involvement in the osteogenic differentiation of human mesenchymal stem cells [33]. In contrast, PDGF receptor signaling regulates mesenchymal stem cell proliferation and migration [34,35], and potentially decreases the expression of collagen type I and bone sialoprotein, which are osteogenic marker genes [33]. Similar results were reported in previous studies when an excessive concentration of the signaling molecules from PRF inhibited or had no effect on mesenchymal stem cell osteogenic differentiation [23,28]. Furthermore, limited osteogenic differentiation has been shown when an enriched platelet product was used to induce mesenchymal stem cell differentiation [23,36,37]. 

The clinical use of i-PRF requires mixing its liquid stage with other particulate biomaterials, and cell differentiation is expected to be the last crucial physiological process occurring inside the bone graft area. Previous results [15] corresponded with ours, showing that the release from red i-PRF during the first 3 d was suitable for recruiting the cells and leading to enhanced PDLSC proliferation, but not osteogenic differentiation. The slightly inferior osteogenic differentiation is likely to increase clinical bone regeneration because it can ensure that an adequate number of cells are recruited to the bone graft site, because healing failed without sufficient mesenchymal stem cells [28]. At the early stage, the wound area can localize more cells or stem cells with the use of red i-PRF, while, at the late stage, a microenvironment appropriate for cell differentiation and tissue formation might be established if the release from red i-PRF declines. However, the therapeutic effects in vivo might be different from the in vitro results. Hence, animal trials, followed by clinical trials, are needed to compare the efficacy and confirm the mechanism.

## 4. Materials and Methods 

### 4.1. Injectable Platelet-Rich Fibrin (i-PRF) Preparation

Blood samples were obtained from five healthy volunteers (age range 30‒35, male/female = 1/4) at the Faculty of Dentistry, Mahidol University (Bangkok, Thailand) after approval by the Institutional Review Board of the Human Ethics Committee of the Faculty of Dentistry, Mahidol University (COA. No. MU-DT/PY-IRB 2017/061.221). All procedures were in accordance with the 1964 Helsinki declaration and its later amendments. The subjects in this study had not taken any antiplatelet or anticoagulant medications. Written consent was obtained before blood collection. Complete blood counts were performed to confirm the overall health status of the subjects, and their results were within normal limits. Sample preparation was performed as described in our previous report [15]. Immediately after the blood sample was collected, the sample was centrifuged at 60 g RCF for 3 min using a Duo Centrifuge (Process for PRF, Nice, France) at room temperature; 60 g RCF is equivalent to 700 rpm for this device that has a 110 mm radius. After centrifugation, two types of i-PRF samples were harvested: yellow and red i-PRF. The yellow i-PRF was the plasma harvested from the upper yellow zone above the erythrocyte–plasma junction, while the red i-PRF was the plasma harvested at the junction with the buffy coat. The beveled edge of the harvesting needle was used as a reference point. The i-PRF was allowed to form a complete clot in 6-well plates (Thermo Fischer Scientific, Waltham, MA, USA) for 30 min before incubation with 5 mL basal medium without fetal bovine serum (FBS; Gibco^TM^, Thermo Fischer Scientific) at 37 °C in a 5% CO_2_ chamber for 72 h. Finally, the medium was aspirated and kept at −20 °C until use as a conditioned media. Twenty percent of the collected medium was added into the basal culture medium or differentiation medium prior to the assays.

### 4.2. PDLSC Isolation and Characterization 

PDLSCs were obtained from another project after approval by the Institutional Review Board of the Human Ethics Committee of the Faculty of Dentistry, Mahidol University (COA. No. MU-DT/PY-IRB 2017/048.0611). Briefly, the PDLSCs were isolated from the middle third of the root surface of healthy premolars extracted for orthodontic reasons. The isolated cells were digested with collagenase type I (3 mg/mL; Worthington Biochemical, Lakewood, NJ, USA) and dispase II (neutral protease, 4 mg/mL; Sigma-Aldrich, St.Louis, MO, USA) for 24 h at 4 °C to obtain a single-cell suspension. Passages 4–6 of the PDLSCs were used and cultured in basal culture medium containing minimum essential medium eagle alpha modification (α-MEM) with nucleosides (Gibco^TM^) supplemented with 10% FBS, 2.2 g/L sodium bicarbonate (Sigma-Aldrich), and 100 units/mL penicillin, 100 ug/mL streptomycin (Gibco^TM^) at 37 °C in a humidified 5% CO_2_ atmosphere. The PDLSCs (3 donor lines) were characterized as mesenchymal stem cells using plastic adherence, cell morphology, and colony-forming units with Giemsa staining. Analysis of the cell surface molecules with a flow cytometer (BD Biosciences, Franklin Lakes, NJ, USA) was performed using positive mesenchymal cell markers, anti-human CD73 (APC/Cy7), anti-human CD90 (PE), anti-human CD105 (Alexa Flour^®^ 488), and anti-human CD146 (PerCP/Cy5.5), together with the negative mesenchymal cell marker anti-human CD34 (APC) (Biolegend, San Diego, CA, USA). Cell multi-lineage differentiation was evaluated using adipogenic, osteogenic, and neurogenic differentiation medium for 28 d with oil red O staining, alizarin red staining, and nestin immunofluorescence, respectively. 

### 4.3. Proliferative Effects of the i-PRF Types on PDLSCs 

Cell proliferation was determined using a 3-(4,5-dimethylthaiazol-2-yl)-2,5-diphenyltetrezolium bromide (MTT) assay. The PDLSCs were plated at a density of 2 × 10^3^ cells/well into 96-well plates 24 h prior to the assay. The culture medium was changed to red or yellow i-PRF conditioned medium for the treatment groups and the basal culture medium for the control group. The medium was changed every 2 d until the end of the experiment. The MTT measurement was done on day 0, 3, and 5 at 570 and 690 nm using a microplate reader (Biotek, Winooski, VT, USA). The proliferation between groups was compared using the average absorbance in each group.

### 4.4. Trans-Well Migration 

Cell migration was performed using a Boyden chamber assay. PDLSCs (2 × 10^4^) in basal medium were seeded into the upper compartment of the polyethylene terephthalate cell culture inserts with an 8 µm pore size (Corning, New York, NY, USA). The cells were allowed to attach for 6 h before changing to serum-free medium for starving the cells. After 12 h of starving, the lower compartment of the well was filled with the red or yellow i-PRF conditioned medium for the treatment groups. Basal culture medium with or without FBS served as a positive and negative control group, respectively. The cells migrated for 24 h before being fixed in 100% methanol for 15 min. Cell staining was performed using 10% Giemsa solution (Merck&Co., Kenilworth, NJ, USA) for 1 h, and the non-migrated cells on the upper side of the membrane were removed using a cotton swab. The images of the migrated cells on the lower side of the membrane were photographed using an Olympus microscope (Olympus, Tokyo, Japan), and the cells were counted with Image J software. The number of migrated cells was statistically compared between groups.

### 4.5. Osteogenic Differentiation 

To compare the potency of the two i-PRFs on osteogenic differentiation, PDLSCs were differentiated into osteoblasts under four different conditions: the two i-PRF treatments and positive and negative controls. PDLSCs (2 × 10^4^) in basal medium were seeded into 24-well culture plates and cultured until 80% confluent. The culture medium was subsequently changed into osteogenic medium (OM), basal medium supplemented with 0.1 µM dexamethasone, 10 mM β-glycerophosphate, and 50 µg/mL ascorbic acid. The treatment groups’ OM was supplemented with either red or yellow i-PRF conditioned medium, while the negative control group comprised only basal medium. The medium was changed every 2 d, and we investigated the PDLSC osteogenic differentiation on day 3 using a quantitative colorimetric measurement of alkaline phosphatase activity (ALP; Abcam, Cambridge, UK) calibrated with total protein quantified with p-nitrophenyl phosphate. Alizarin red staining of the calcified tissue was analyzed on days 7, 14, and 21. The stained calcium was examined using a light microscope (Olympus, Tokyo, Japan). Images were obtained with a Canon EOS 5D camera, and the alizarin red quantification was performed using ImageJ software as previously described [38]. The color intensity was measured using the same parameter settings and threshold values. Three 40× magnification images of the center area were randomly selected from each well, and the average was used for statistical analysis.

### 4.6. Statistical Analysis 

Statistical assessment was performed using SPSS version 18.0. The results are presented as the mean value ± standard deviation. The cell proliferation, migration, and differentiation assays were performed in triplicate for each cell line, and the data were analyzed by one-way analysis of variance followed by post hoc least-significant difference tests to compare the differences between groups in each experiment. A *p*-value < 0.05 was considered significant.

## 5. Conclusions

Our study provides in vitro evidence of different biological effects of the i-PRF from different fractions. The red i-PRF mobilizes healing-associated stem cells and promotes better proliferation of the mobilized cells, which is beneficial for early-stage wound healing and bone regeneration. Furthermore, the red i-PRF is not likely to impede bone regeneration or induce premature bone formation outside the desire area. Thus, the use of red i-PRF combined with a bone substitute material may be superior to the yellow i-PRF.

## Abbreviations

i-PRF	Injectable-platelet rich fibrin
PDLSCs	Periodontal ligament stem cells
PDGF	Platelet-derived growth factor
VEGF	Vascular endothelial growth factors
TGF-β1	Transforming growth factor-beta 1
OM	Osteogenic medium

## References

[1] Jung R.E., Schmoekel H.G., Zwahlen R., Kokovic V., Hammerle C.H., Weber F.E. (2005). Platelet-rich plasma and fibrin as delivery systems for recombinant human bone morphogenetic protein-2. Clin. Oral Implants Res..

[2] Chotitumnavee J., Parakaw T., Srisatjaluk R.L., Pruksaniyom C., Pisitpipattana S., Thanathipanont C., Amarasingh T., Tiankhum N., Chimchawee N., Ruangsawasdi N. (2019). In vitro evaluation of local antibiotic delivery via fibrin hydrogel. J. Dent. Sci..

[3] Spotnitz W.D. (2014). Fibrin Sealant: The Only Approved Hemostat, Sealant, and Adhesive-a Laboratory and Clinical Perspective. ISRN Surg..

[4] Dohan D.M., Choukroun J., Diss A., Dohan S.L., Dohan A.J., Mouhyi J., Gogly B. (2006). Platelet-rich fibrin (PRF): A second-generation platelet concentrate. Part I: Technological concepts and evolution. Oral Surg. Oral Med. Oral Pathol. Oral Radiol. Endod..

[5] Shah R., Gowda T.M., Thomas R., Kumar T., Mehta D.S. (2019). Biological activation of bone grafts using injectable platelet-rich fibrin. J. Prosthet. Dent..

[6] Ghanaati S., Booms P., Orlowska A., Kubesch A., Lorenz J., Rutkowski J., Landes C., Sader R., Kirkpatrick C., Choukroun J. (2014). Advanced platelet-rich fibrin: A new concept for cell-based tissue engineering by means of inflammatory cells. J. Oral Implantol..

[7] Abd El Raouf M., Wang X., Miusi S., Chai J., Mohamed AbdEl-Aal A.B., Nefissa Helmy M.M., Ghanaati S., Choukroun J., Choukroun E., Zhang Y. (2019). Injectable-platelet rich fibrin using the low speed centrifugation concept improves cartilage regeneration when compared to platelet-rich plasma. Platelets.

[8] Kyyak S., Blatt S., Pabst A., Thiem D., Al-Nawas B., Kammerer P.W. (2020). Combination of an allogenic and a xenogenic bone substitute material with injectable platelet-rich fibrin—A comparative in vitro study. J. Biomater. Appl..

[9] Mourao C.F., Valiense H., Melo E.R., Mourao N.B., Maia M.D. (2015). Obtention of injectable platelets rich-fibrin (i-PRF) and its polymerization with bone graft: Technical note. Rev. Col. Bras. Cir..

[10] Gulsen U., Dereci O. (2019). Evaluation of New Bone Formation in Sinus Floor Augmentation With Injectable Platelet-Rich Fibrin-Soaked Collagen Plug: A Pilot Study. Implant Dent..

[11] Xie H., Xie Y.F., Liu Q., Shang L.Y., Chen M.Z. (2019). Bone regeneration effect of injectable-platelet rich fibrin (I-PRF) in lateral sinus lift: A pilot study. Shanghai Kou Qiang Yi Xue.

[12] Ozsagir Z.B., Saglam E., Sen Yilmaz B., Choukroun J., Tunali M. (2020). Injectable platelet-rich fibrin and microneedling for gingival augmentation in thin periodontal phenotype: A randomized controlled clinical trial. J. Clin. Periodontol..

[13] Ucak Turer O., Ozcan M., Alkaya B., Surmeli S., Seydaoglu G., Haytac M.C. (2020). Clinical evaluation of injectable platelet-rich fibrin with connective tissue graft for the treatment of deep gingival recession defects: A controlled randomized clinical trial. J. Clin. Periodontol..

[14] Wang X., Zhang Y., Choukroun J., Ghanaati S., Miron R.J. (2018). Effects of an injectable platelet-rich fibrin on osteoblast behavior and bone tissue formation in comparison to platelet-rich plasma. Platelets.

[15] Thanasrisuebwong P., Surarit R., Bencharit S., Ruangsawasdi N. (2019). Influence of Fractionation Methods on Physical and Biological Properties of Injectable Platelet-Rich Fibrin: An Exploratory Study. Int. J. Mol. Sci..

[16] Kim S.H., Kim K.H., Seo B.M., Koo K.T., Kim T.I., Seol Y.J., Ku Y., Rhyu I.C., Chung C.P., Lee Y.M. (2009). Alveolar bone regeneration by transplantation of periodontal ligament stem cells and bone marrow stem cells in a canine peri-implant defect model: A pilot study. J. Periodontol..

[17] Miron R.J., Fujioka-Kobayashi M., Hernandez M., Kandalam U., Zhang Y., Ghanaati S., Choukroun J. (2017). Injectable platelet rich fibrin (i-PRF): Opportunities in regenerative dentistry?. Clin. Oral Investig..

[18] Varela H.A., Souza J.C.M., Nascimento R.M., Araujo R.F., Vasconcelos R.C., Cavalcante R.S., Guedes P.M., Araujo A.A. (2019). Injectable platelet rich fibrin: Cell content, morphological, and protein characterization. Clin. Oral Investig..

[19] Miron R.J., Chai J., Zheng S., Feng M., Sculean A., Zhang Y. (2019). A novel method for evaluating and quantifying cell types in platelet rich fibrin and an introduction to horizontal centrifugation. J. Biomed. Mater. Res. A.

[20] Donos N., Dereka X., Calciolari E. (2019). The use of bioactive factors to enhance bone regeneration: A narrative review. J. Clin. Periodontol..

[21] Fernandez-Medina T., Vaquette C., Ivanovski S. (2019). Systematic Comparison of the Effect of Four Clinical-Grade Platelet Rich Hemoderivatives on Osteoblast Behaviour. Int. J. Mol. Sci..

[22] Gassling V.L., Acil Y., Springer I.N., Hubert N., Wiltfang J. (2009). Platelet-rich plasma and platelet-rich fibrin in human cell culture. Oral Surg. Oral Med. Oral Pathol. Oral Radiol. Endod..

[23] Jin R., Song G., Chai J., Gou X., Yuan G., Chen Z. (2018). Effects of concentrated growth factor on proliferation, migration, and differentiation of human dental pulp stem cells in vitro. J. Tissue Eng..

[24] Chen L., Tredget E.E., Wu P.Y., Wu Y. (2008). Paracrine factors of mesenchymal stem cells recruit macrophages and endothelial lineage cells and enhance wound healing. PLoS ONE.

[25] Shirley D., Marsh D., Jordan G., McQuaid S., Li G. (2005). Systemic recruitment of osteoblastic cells in fracture healing. J. Orthop. Res..

[26] Su P., Tian Y., Yang C., Ma X., Wang X., Pei J., Qian A. (2018). Mesenchymal Stem Cell Migration during Bone Formation and Bone Diseases Therapy. Int. J. Mol. Sci..

[27] Bhattacharya I., Ghayor C., Weber F.E. (2016). The Use of Adipose Tissue-Derived Progenitors in Bone Tissue Engineering—A Review. Transfus. Med. Hemother..

[28] Lienemann P.S., Vallmajo-Martin Q., Papageorgiou P., Blache U., Metzger S., Kivelio A.S., Milleret V., Sala A., Hoehnel S., Roch A. (2020). Smart Hydrogels for the Augmentation of Bone Regeneration by Endogenous Mesenchymal Progenitor Cell Recruitment. Adv. Sci. (Weinh).

[29] Moisley K.M., El-Jawhari J.J., Owston H., Tronci G., Russell S.J., Jones E.A., Giannoudis P.V. (2019). Optimising proliferation and migration of mesenchymal stem cells using platelet products: A rational approach to bone regeneration. J. Orthop. Res..

[30] Vander Heiden M.G., Plas D.R., Rathmell J.C., Fox C.J., Harris M.H., Thompson C.B. (2001). Growth factors can influence cell growth and survival through effects on glucose metabolism. Mol. Cell Biol..

[31] Somerman M.J., Young M.F., Foster R.A., Moehring J.M., Imm G., Sauk J.J. (1990). Characteristics of human periodontal ligament cells in vitro. Arch. Oral Biol..

[32] Winning L., El Karim I.A., Lundy F.T. (2019). A Comparative Analysis of the Osteogenic Potential of Dental Mesenchymal Stem Cells. Stem Cells Dev..

[33] Kumar A., Salimath B.P., Stark G.B., Finkenzeller G. (2010). Platelet-derived growth factor receptor signaling is not involved in osteogenic differentiation of human mesenchymal stem cells. Tissue Eng. Part A.

[34] Ponte A.L., Marais E., Gallay N., Langonne A., Delorme B., Herault O., Charbord P., Domenech J. (2007). The in vitro migration capacity of human bone marrow mesenchymal stem cells: Comparison of chemokine and growth factor chemotactic activities. Stem Cells.

[35] Fierro F., Illmer T., Jing D., Schleyer E., Ehninger G., Boxberger S., Bornhäuser M. (2007). Inhibition of platelet-derived growth factor receptorbeta by imatinib mesylate suppresses proliferation and alters differentiation of human mesenchymal stem cells in vitro. Cell Prolif..

[36] Gruber R., Karreth F., Kandler B., Fuerst G., Rot A., Fischer M.B., Watzek G. (2004). Platelet-released supernatants increase migration and proliferation, and decrease osteogenic differentiation of bone marrow-derived mesenchymal progenitor cells under in vitro conditions. Platelets.

[37] Vogel J.P., Szalay K., Geiger F., Kramer M., Richter W., Kasten P. (2006). Platelet-rich plasma improves expansion of human mesenchymal stem cells and retains differentiation capacity and in vivo bone formation in calcium phosphate ceramics. Platelets.

[38] Miron R.J., Fujioka-Kobayashi M., Zhang Y., Sculean A., Pippenger B., Shirakata Y., Kandalam U., Hernandez M. (2017). Osteogain(R) loaded onto an absorbable collagen sponge induces attachment and osteoblast differentiation of ST2 cells in vitro. Clin. Oral Investig..

